# Two phase 1 studies of safety, tolerability, and pharmacokinetics of an EFdA prodrug (BRII-732) in healthy adult participants

**DOI:** 10.1128/aac.00200-25

**Published:** 2025-04-22

**Authors:** David Margolis, Michael Watkins, Yali Zhu

**Affiliations:** 1 Brii Biosciences Inc. 733165 , Durham, North Carolina, USA; IrsiCaixa Institut de Recerca de la Sida, Barcelona, Spain

**Keywords:** antiviral agents, phase 1, pharmacokinetics, safety, EFdA, BRII-732

## Abstract

BRII-732, a medoxomil carbonate prodrug of EFdA, is designed for once weekly dosing as part of combination antiretroviral therapy for HIV. Two single-center, randomized, double-blind, placebo-controlled phase 1 studies evaluated its safety, tolerability, and pharmacokinetics in 72 healthy adult participants. Study 1 assessed single doses (10–200 mg) and multiple weekly doses (10, 25 mg), while Study 2 investigated lower doses (≤2.5 mg) and relative bioavailability of tablet versus oral solution. In Study 1, the first single-dose cohort and the first multiple-dose cohort each enrolled four participants (three active; one placebo); the remaining cohorts each enrolled eight participants (six active; two placebo). In Study 2, each cohort enrolled eight participants (six active; two placebo). BRII-732 was well tolerated, with most TEAEs being mild and no SAEs, or withdrawals. Clinical laboratory tests, vital signs, and ECGs revealed no significant abnormalities. Plasma BRII-732 concentrations were largely below the limit of quantification, confirming efficient conversion to EFdA. Plasma exposures of EFdA increased near dose-proportionality, with a mean *t*_1/2_ of 1.50–2.94 hours (≤10 mg) and 55–112 hours (25–200 mg). EFdA showed no accumulation in plasma after weekly dosing. Intracellularly, EFdA-TP demonstrated rapid formation, slow elimination (*t*_1/2_: 194–227 hours), and meaningful accumulation after weekly dosing, with an estimated steady-state accumulation ratio of ~2.2–2.5. Tablet and solution formulations exhibited comparable systemic exposure. These studies highlight the favorable safety, tolerability, and pharmacokinetic profile of once weekly BRII-732 in healthy adult participants.

## INTRODUCTION

Effective combination antiretroviral therapy (cART) treatment of human immunodeficiency virus (HIV) infection changes the natural history of the infection, allowing for a normal life expectancy for those who are adherent to therapy both in developed and developing countries, transforming HIV infection into a manageable chronic disease. Once daily single tablet regimens (STR) improved adherence and patient satisfaction, compared with regimens requiring multiple pills and higher dosing frequency. However, HIV patients desire further improvement in quality of life (QoL) and seek less frequent treatment options that remove daily reminders of their disease status while providing freedom to engage in activities without having to carry HIV medicines (1, 2).

Long-acting therapy (LAT), dosed once a month or once every 2 months, maintains efficacious drug levels through slow release of the drug from a depot deposited via injection (3–5). Monthly intramuscular injection of cabotegravir/rilpivirine (400 mg/600 mg) demonstrated non-inferior efficacy and safety compared to current antiretroviral oral regimens in two phase 3 studies (ATLAS and FLAIR, up to 96 week maintenance therapy) (6–10). For many participants, improvement of QoL and freedom from the need for daily oral therapy appear to be a major advance, even at the cost of requiring monthly injections in the clinic.

A patient survey study showed that given the choice of LATs, a single oral pill once weekly was preferred by a majority of patients over the administration of two injections in the clinic every other month or implantation and removal of two small plastic rods every 6 months (11). Therefore, a once weekly single tablet regimen may offer an important new treatment option for people living with HIV who seek a better QoL without the daily reminders of their HIV status and who desire a more discreet, non-invasive way of taking their medication.

BRII-732 is a medoxomil carbonate prodrug of 4′-ethynyl-2-fluoro-2′-deoxyadenosine (EFdA). Similar to other medoxomil-based prodrugs, it undergoes rapid enzymatic hydrolysis, likely by esterases and phosphatases, both extracellularly and intracellularly during or shortly after absorption. Current development work with BRII-732 is designed to provide optimal drug exposure to enable once weekly dosing as part of cART therapy while improving patient convenience, QoL, and treatment adherence. During the course of BRII-732 clinical development, a clinical trial observation of decreasing total lymphocytes and CD4+ T cell counts in a dose-dependent manner was identified with islatravir, or EFdA, resulting in FDA clinical trial holds across multiple islatravir and islatravir-related studies. Consequently, FDA placed BRII-732 on clinical hold as the active moiety of BRII-732 is also EFdA. Clinical trials of EFdA and BRII-732 were allowed to resume based on observed and modeled data that demonstrated that islatravir and intracellular islatravir triphosphate concentrations associated with oral doses of islatravir 0.25 mg once daily and/or 2 mg once weekly achieved similar CD4+ T cell and lymphocyte dynamics compared to standard ART (12).

This paper reports results from two phase 1 studies that assessed the safety, tolerability, and pharmacokinetics (PK) of BRII-732 in healthy adult participants. Study 1 evaluated single doses up to 200 mg and multiple doses up to 25 mg BRII-732 prior to FDA clinical hold, while Study 2 evaluated a lower dose option, single doses up to 2.5 mg, following FDA lift of the clinical hold.

## MATERIALS AND METHODS

### Study design

Both Study 1 and Study 2 were phase 1, randomized, double-blind, placebo-controlled, single ascending dose (SAD) and multiple ascending dose (MAD) single-center studies to assess safety, tolerability, and pharmacokinetics of BRII-732 in healthy adult participants. Study 1 was the first-in-human study of BRII-732 administered as an oral solution. The objective of Study 2 was to identify a once weekly therapeutic dose with an acceptable safety profile, avoiding an impact on total lymphocyte and CD4+ T cell counts, using a tablet formulation of BRII-732.

Study 1 consisted of 5 SAD cohorts and 2 MAD cohorts. Eligible participants had a screening visit within 28 days prior to BRII-732 administration on Day 1. Participants in the SAD cohorts remained as inpatients at the clinical site until Day 14 and attended an outpatient visit on Day 28. Participants in the MAD cohorts remained as inpatients at the clinical site until Day 28, were contacted by phone on Day 35, and attended an outpatient visit on Day 42. BRII-732 was supplied as a solution prepared by the clinic pharmacy for oral administration in the fasted state. After each cohort, a Safety Review Committee (SRC), in accordance with the SRC charter, performed reviews of safety and tolerability data collected for dose-escalation decisions and to guide the progress of the study, ensuring the safety of the participants. During the SAD phase of the study, cohort dosing of 10, 25, 50, 100, and 200 mg of BRII-732 was evaluated in a sequential manner. Two sentinel participants were administered study drug (one BRII-732 and one placebo) and were monitored and assessed for safety for 4–6 hours postdose to determine if the remainder of the cohort could be dosed on Day 1 based on review of safety data. The first SAD cohort enrolled four participants (three active; one placebo); each of the remaining four SAD cohorts enrolled eight eligible participants (six active; two placebo). During the MAD phase of the study, 10 and 25 mg of BRII-732 administered once weekly for total three doses were evaluated in sequential cohorts comprised of four participants (three active; one placebo) for the first MAD cohort and eight participants (six active; two placebo) in the subsequent cohort.

Study 2 consisted of three SAD cohorts. Eligible participants had a screening visit within 28 days prior to BRII-732 administration on Day 1. Participants remained as inpatients at the clinical site until Day 14 and attended an outpatient visit on Days 20 and 28. BRII-732 was supplied as a 1 mg and 2.5 mg solution prepared by the clinic pharmacy (cohorts 1 and 3, respectively) and as 2.5 mg tablets (cohort 2) for oral administration in the fasted state. An SRC reviewed safety and tolerability data after cohort 1 to determine dose escalation to cohorts 2 and 3.

Eligible study participants were male or female of non-childbearing potential, aged 18–55 years, and body mass index was within the range of 18.5 and 32 kg/m^2^. Participants were deemed medically healthy with clinically insignificant screening results by the principal investigator (e.g., laboratory profiles, medical histories, electrocardiograms [ECGs], and physical examination). Participants were excluded if they had positive testing for HIV-1 or HIV-2, hepatitis A antibody, hepatitis B surface antigen and/or core antibody, hepatitis C virus, or COVID rapid PCR, an excessive history of alcohol intake (more than two drinks for women or four drinks for men per day), received any vaccine or prescription medication within 14 days (or 5 half-lives) prior to day 1, use of over-the-counter medication within 7 days prior to day 1, had a CD4 count below the normal range (<500 cell/mL^3^), total lymphocyte count below the normal range, eGFR less than 80 mL/min/1.73 m^2^, or Fridericia’s corrected QT interval greater than 440 ms.

Both studies were conducted in accordance with Good Clinical Practice guidelines, including the International Conference on Harmonization of Technical Requirements for Registration of Pharmaceuticals for Human Use guidelines, and applicable regulatory requirements outlined in the Declaration of Helsinki. Written informed consent was obtained from each participant prior to any study-related activities. The study protocol and informed consent form were reviewed and approved by an independent review board.

### Safety

For both studies, safety assessments included repeated measures of laboratory testing, vital signs, ECGs, physical examinations, and adverse event (AE) monitoring and reporting conducted before, during, and after study drug administration. Study 2 also included additional lymphocyte testing, including cell differentiation and CD4+ and CD8+ subset testing at screening and serially throughout the study.

### Pharmacokinetic assessments

Blood samples for analyses of BRII-732 and EFdA in plasma and intracellular metabolite EFdA-TP in PBMC were obtained at predefined timepoints.

#### Study 1 SAD cohorts

Before dosing and at 0.5, 1, 4, 7, 12, 24, 48, 72, 96, 120, 144, 168, 192, 216, 264, 312, and 648 hours post-dose.

#### Study 1 MAD cohorts

Before dosing and at 0.5, 1, 4, 7, 12, 24, 48, 72, 96, 120, 144, and 168 hours after the first dose (Day 1) and the last dose (Day 15). Additional samples at 192, 216, 264, 312, and 648 hours post-dose were also collected after the last dose on Day 15.

#### Study 2 SAD cohorts

Before dosing and at 0.5, 1, 2, 4, 6, 8, 12 (BRII-732 and EFdA only), 24, 48, 72 (EFdA-TP only), 96 (BRII-732 and EFdA only), 120 (EFdA-TP only), and 168 hours post-dose. Additional samples at 216, 312, 456, and 648 hours post-dose were also collected for analysis of EFdA-TP in PBMCs.

For plasma samples, blood was collected into a 4 mL tube containing K2EDTA and then added 200 µL of inhibitor cocktail solution (roughly 19:1 ratio to the blood volume), immediately inverted 10–12 times and placed on ice, and centrifuged at 1,500 × *g* for approximately 10 minutes at 2–8°C. Supernatant plasma was transferred to two cryovials (ca. 400 mL per vial) and stored at −70°C until shipped to the bioanalytical laboratory for the determination of BRII-732 and EFdA concentrations using validated high-performance liquid chromatography-tandem mass spectrometry (LC-MS/MS) methods. The assay range was 1.00–1,000 ng/mL for BRII-732 and 1.00–3,000 ng/mL for EFdA. The assay precision for BRII-732 and EFdA was 5.4%–9.7% and 6.2%–12.3%, respectively, and accuracy was 97.7%–103.2% and 96.4%–101.7%, respectively.

For PBMC samples, blood was collected into 8 mL Cell Preparation Tubes (CPT) containing sodium heparin, invert 8–10 times and kept at room temperature, centrifuged at 1,500–1,800 × *g* at ambient temperature for at least 15 minutes. After aspirating the plasma, the PBMCs were washed twice with PBS buffer and then washed and resuspended in RPMI-1640 medium. The cell count was performed, and lysis buffer was added based on cell counts per sample. The final PBMC lysate was stored at − 70°C until shipped to the bioanalytical laboratory for the determination of EFdA-TP concentrations using validated LC-MS/MS methods. The assay range was 0.100–40.0 ng/mL, precision was 1.4%–10.5%, and accuracy was 98%–103%.

The analyses of both plasma and PBMC samples were conducted at Alliance Pharma (now Resolian). All Incurred Sample Reanalysis (ISR) met acceptance criteria.

### Pharmacokinetic data analyses

BLOQ Concentrations were not imputed. BLOQ values occurring before the first quantifiable concentration were assigned a value of zero. Thereafter, BLOQ concentrations were treated as missing. For the calculation of mean concentrations, these missing values were included.

Area under the concentration-time curve (AUC) from time zero to the time of the last quantifiable concentration (AUC_0-last_), AUC from time zero to infinity (AUC_0-inf_), AUC within a dosing interval of 168 hours (AUC_0-168h_), maximum observed concentration (*C*_max_), time to reach maximum concentration (*T*_max_), concentration at end of dosing interval (*C*_168h_), apparent terminal half-life (*t*_1/2_), apparent total clearance (CL/F), apparent volume of distribution during terminal phase (Vz/F), and accumulation ratio based on AUC (AR_AUC_) were determined. PK parameters were determined using noncompartmental methods in Phoenix WinNonlin (Certara, Version 8.3) and summarized for each cohort using descriptive statistics.

To assess dose proportionality in Study 1, PK exposure parameters were natural log-transformed and statistically compared between dose levels using a mixed effects model including dose level as a fixed effect. To assess the relative bioavailability of BRII-732 oral tablet versus solution in Study 2, an analysis of variance model was used for natural log-transformed PK exposure parameters; the model included treatment (formulation) as a factor. For both statistical analyses, geometric least-squares means (GLSMs), ratios of GLSMs, and corresponding 90% CIs were constructed.

## RESULTS

### Participant characteristics

For Study 1, all participants (*N* = 48) who were randomized and dosed completed the study, and no participants were discontinued. Mean age of participants was 38 years in the SAD cohorts and 39 years in the MAD cohorts. A majority of participants in Study 1 were female (SAD: 61% and MAD: 50%) and white (SAD: 56% and MAD: 58%) (Table 1). For Study 2, 24 participants were randomized and dosed: 22 participants completed the study, and 2 participants withdrew by choice. Mean age of participants in the SAD cohorts was 34 years and a majority of participants were male (71%) and white (67%) (Table 2). For either study, no notable differences were observed among treatment groups or between participants who received BRII-732 and those who received placebo with respect to demographic parameters.

**TABLE 1:** Participant demographics and baseline characteristics in Study 1 SAD and MAD cohorts^*a*^

SAD cohorts							MAD cohorts		
		BRII-732						BRII-732	
Parameter	Placebo solution	10 mg solution	25 mg solution	50 mg solution	100 mg solution	200 mg solution	Placebo solution	10 mg solution QW X 3	25 mg solution QW X 3
No. randomized	9	3	6	6	6	6	3	3	6
Age (years)	36 ± 12	35 ± 9	35 ± 7	43 ± 9	41 ± 8	36 ± 6	40 ± 11	40 ± 11	38 ± 6
Female (%)	6 (67)	2 (67)	2 (33)	6 (100)	4 (67)	2 (33)	1 (33)	2 (67)	3 (50)
White (%)	6 (67)	2 (67)	1 (17)	6 (100)	3 (50)	2 (33)	3 (100)	0	4 (67)
Height (cm)	164 ± 12	163 ± 20	171 ± 8	158 ± 4	163 ± 8	170 ± 10	168 ± 8	165 ± 3	170 ± 11
Weight (kg)	67 ± 16	73 ± 9	81 ± 13	66 ± 3	73 ± 12	77 ± 8	76 ± 12	74 ± 9	80 ± 12
BMI (kg/m^2^)	25 ± 3	28 ± 4	28 ± 3	27 ± 1	27 ± 3	27 ± 3	27 ± 4	27 ± 4	28 ± 3.5

^*a*^ BMI, body mass index; *N*, number of participants; MAD, multiple ascending dose; SAD, single ascending dose. Mean ± Standard deviation is presented for continuous variables (age, height, weight, and BMI); *N* (%) is presented for categorical variables (female and White).

**TABLE 2:** Participant demographics and baseline characteristics in Study 2 SAD cohorts^*a*^

				BRII-732	
Parameter	Placebo solution	Placebo tablet	1 mg solution	2.5 mg tablet	2.5 mg solution
No. randomized	4	2	6	6	6
Age (years)	37.0 ± 5	32 ± 13	33 ± 7	32 ± 9	36 ± 11
Female (%)	1 (25)	—^*b*^	2 (33)	2 (33)	2 (33)
White (%)	3 (75)	2 (100)	2 (33.3)	4 (67)	5 (83)
Height (cm)	172 ± 6	174 ± 13	167 ± 10	171 ± 6	174 ± 9
Weight (kg)	84 ± 10	73 ± 8	69 ± 14	76 ± 12	78 ± 15
BMI (kg/m^2^)	28 ± 2	24 ± 1	25 ± 5	26 ± 4	26 ± 3

^*a*^ BMI, body mass index; *N,* number of participants; SAD, single ascending dose. Mean ± Standard deviation is presented for continuous variables (age, height, weight, and BMI); *N* (%) is presented for categorical variables (female and White).

^*b*^ "—", Not Available.

### Safety

In Study 1, 12 of 36 (33.3%) participants in the SAD cohorts reported 1 or more TEAEs, 3 of which were considered related to BRII-732 by the investigator. No TEAEs were reported following dosing with the lowest dose (10 mg) and the highest dose (200 mg) of BRII-732 solution. The majority of reported TEAEs were mild (Grade 1) in severity (83.3%) with a single participant reporting a moderate (Grade 2) TEAE in the pooled placebo group and a single participant who received a single dose of 100 mg of BRII-732 reporting a severe (Grade 3) increased low-density lipoprotein TEAE and a moderate (Grade 2) increased alanine aminotransferase TEAE. The only TEAEs reported by more than one participant in the SAD cohorts were constipation and headache. In the MAD cohorts, 7 (58.3%) participants had 1 or more TEAEs which were all of mild severity. Six participants had TEAEs that were considered by the Investigator to be related to study drug. The most common TEAE reported in the MAD cohorts was nausea (*n* = 4). All TEAEs for Study 1 had resolved by the end of the study. There were no deaths, SAEs, or TEAEs leading to study withdrawal.

In Study 2, 5 of 24 (20.8%) participants reported 5 TEAEs, 2 of which were considered related to BRII-732 by the investigator. The majority were mild (Grade 1) in severity, with one of the five TEAEs reported by one participant that was a moderate (Grade 2) TEAE of trichomoniasis. No TEAEs were reported following dosing with the 1 mg BRII-732 solution or the 2.5 mg BRII-732 tablet. All TEAEs had resolved by the end of the study. There were no deaths, SAEs, or TEAEs leading to study withdrawal.

### Clinical laboratory tests, vital signs, ECG measurements

In Study 1, the majority of laboratory abnormalities were DAIDs Grade 1. Grade 3 LDL increases were reported for one participant (33.3%) receiving a single dose of 10 mg BRII-732 oral solution and for one participant (16.7%) receiving a single dose of 100 mg BRII-732 oral solution compared to one participant (11.1%) receiving a single dose of placebo. No graded lymphocyte decreases were reported. No participants developed lymphopenia of clinical concern or lymphocyte-related AE.

In Study 2, there were no clinically meaningful results or trends in clinical laboratory evaluations including no treatment- or dose-related trends in the mean or individual participant serum biochemistry, hematology, and lymphocyte CD4+/CD8+ subsets during the study. There were no reports of laboratory results that were Grade ≥ 3. For both studies, no safety issues were observed with respect to vital sign results or ECG measurements.

### Pharmacokinetics

#### BRII-732 in plasma

After single oral administration of BRII-732 at doses up to 200 mg, BRII-732, a prodrug, was efficiently converted to EFdA. Plasma concentrations of BRII-732 remained below the limit of quantification (BLOQ) of 1.00 ng/mL throughout the sample collection duration of up to 648 hours post-dose. Thus, no PK results are presented for BRII-732.

Similarly, after three weekly oral administrations of BRII-732 at doses of 10 mg and 25 mg, BRII-732 plasma concentrations were mostly BLOQ throughout the sample collection duration, except for a few low (<15 ng/mL) sporadic measurable concentrations in one participant at 10 mg dose and three participants at 25 mg dose. No PK parameters were calculated for BRII-732.

#### EFdA in plasma

**Fig 1:**
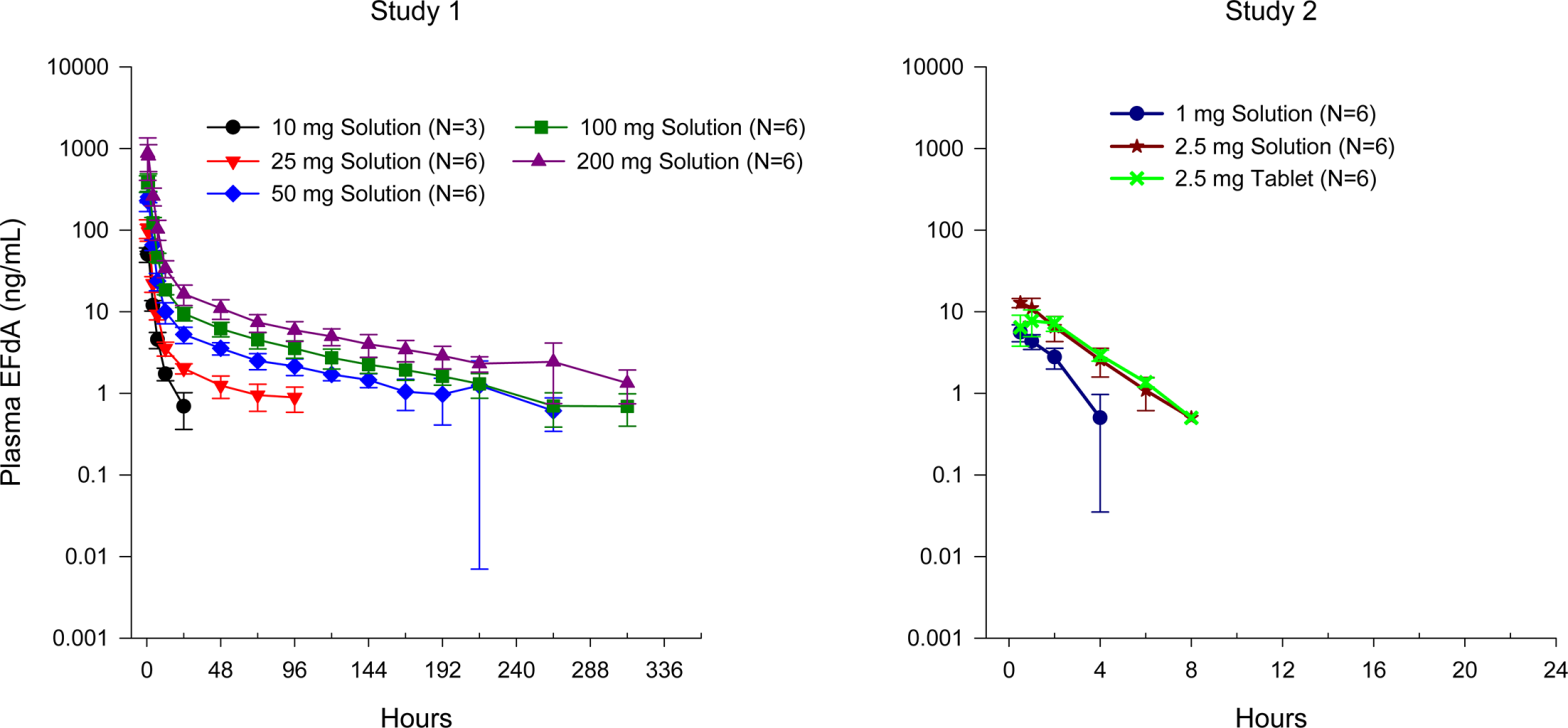
Mean (±SD) plasma EFdA concentrations following single ascending doses of BRII-732.

After single oral administration of BRII-732, plasma concentration profiles of EFdA indicated rapid absorption of BRII-732 and subsequent hydrolytic release of EFdA. Maximum EFdA plasma concentrations were achieved at median *T*_max_ of 0.50–1.00 hours (Table 3). Following *C*_max_, EFdA plasma concentrations exhibited initial rapid decline, followed by slower disposition phases (Fig. 1). The elimination *t*_1/2_ at 1 mg dose could not be calculated due to limited quantifiable concentrations during the terminal phase. The mean *t*_1/2_ was 1.50–2.94 hours after single doses up to 10 mg and ranged from 55 to 112 hours over the dose range of 25–200 mg. The long *t*_1/2_ at higher dose levels was primarily driven by its sustained low concentrations over an extended period of time.

**TABLE 3:** EFdA mean pharmacokinetic parameters in plasma following single ascending doses of BRII-732^*a*^

	Study 1					Study 2		
Parameter	10 mg solution (*N* = 3)	25 mg solution (*N* = 6)	50 mg solution (*N* = 6)	100 mg solution (*N* = 6)	200 mg solution (*N* = 6)	1 mg solution (*N* = 6)	2.5 mg solution (*N* = 6)	2.5 mg tablet (*N* = 6)
*C*_max_ (ng/mL)	55.6 (8)	112 (21)	265 (14)	401 (23)	986 (32)	5.66 (22)	13.9 (7)	8.81 (17)
*T*_max_ (h)	1.0 (0.5, 1.0)	0.5 (0.5, 1.0)	0.75 (0.5, 1.0)	1.0 (0.5, 1.02)	0.5 (0.5, 1.0)	0.5 (0.5, 1.0)	0.5 (0.5, 1.0)	1.0 (0.5, 2.0)
AUC_0-last_ (h*ng/mL)	164 (10)	406 (20)	1,287 (19)	2,244 (19)	4,580 (26)	9.59 (35)	29.1 (31)	25.8 (12)
AUC_0-inf_ (h*ng/mL)	168 (14)^*b*^	491 (32)^*c*^	1,459 (17)^*d*^	2,393 (17)	4,652 (33)^*c*^	NC	32.3 (29)	30.3 (8)^*c*^
*t*_1/2_ (h)	2.94 (0.48)^*b*^	54.9 (87)^*c*^	112 (9)^*d*^	87.0 (12)	97.3 (6)^*c*^	NC	1.50 (11)	1.74 (20)^*c*^

^*a*^ Data are presented as mean (CV%); for *T*_max_, median (min, max) is reported. NC, not calculable.

^*b*^ n=2.

^*c*^ n=4.

^*d*^ n=5.

After single doses in Study 1, EFdA plasma exposures (*C*_max_ and AUCs) showed dose-dependent increases. As shown in Table 4, using exposure values at 10 mg as a reference, GLSM-based statistical comparison indicated near dose-proportional increases in exposure over the dose range of 10–200 mg. At the same dose level of 2.5 mg BRII-732 in Study 2, mean *C*_max_ of EFdA for oral tablet was lower than that for solution, but overall systemic exposure (AUCs) was comparable between tablet and solution formulations. Statistical analysis showed that the ratio of GLSMs was 62.8% for *C*_max_ with the upper bound of 90% CI below 100% and was 92.4% and 97.5% for AUC_0-last_ and AUC_0-inf_, respectively, with 90% CIs spanned 100% (Table 5).

**TABLE 4:** Statistical comparisons of EFdA pharmacokinetic parameters between dose levels (Study 1)^*a*^

Parameters	BRII-732 dose	*n*	GLSM	Test versus reference GLSM ratio (90% CI)
*C*_max_ (ng/mL)	10 mg (reference)	3	55.5	—^*b*^
	25 mg (test)	6	109	1.97 (1.49, 2.60)
	50 mg (test)	6	262	4.73 (3.59, 6.24)
	100 mg (test)	6	391	7.04 (5.33, 9.28)
AUC_0-168h_ (h*ng/mL)	10 mg (reference)	2	168	—
	25 mg (test)	4	432	2.58 (1.85, 3.59)
	50 mg (test)	6	1,227	7.32 (5.35, 10.0)
	100 mg (test)	6	2,103	12.6 (9.18, 17.2)
AUC_0-last_ (h*ng/mL)	10 mg (reference)	3	163	—
	25 mg (test)	6	397	2.43 (1.87, 3.15)
	50 mg (test)	6	1,267	7.76 (5.98, 10.1)
	100 mg (test)	6	2,208	13.5 (10.4, 17.5)

^*a*^ CI = confidence interval; GLSM = geometric least square mean.

^*b*^ "—", Not Appropriate.

**TABLE 5:** Statistical analysis for relative bioavailability of BRII-732 oral tablet (Study 2)^*a*^

Analyte	Parameters	BRII-732 dose	*n*	GLSM	Test versus reference percent GLSM ratio (90% CI)
EFdA	*C*_max_ (ng/mL)	2.5 mg Solution (reference)	6	13.8	—^*b*^
		2.5 mg Tablet (test)	6	8.68	62.8 (54.1, 73.1)
	AUC_0-last_ (h*ng/mL)	2.5 mg Solution (reference)	6	27.8	—
		2.5 mg Tablet (test)	6	25.7	92.4 (70.0, 122)
	AUC_0-inf_ (h*ng/mL)	2.5 mg Solution (reference)	6	31.0	—
		2.5 mg Tablet (test)	4	30.2	97.5 (71.1, 134)
EFdA-TP	*C*_max_ (pmol/10^6^ cells)	2.5 mg Solution (reference)	6	2.75	—
		2.5 mg Tablet (test)	6	2.58	94.0 (65.4, 135)
	AUC_0-last_ (h*pmol/10^6^ cells)	2.5 mg Solution (reference)	6	408	—
		2.5 mg Tablet (test)	6	360	88.3 (65.3, 119)
	AUC_0-inf_ (h*pmol/10^6^ cells)	2.5 mg Solution (reference)	6	455	—
		2.5 mg Tablet (test)	3	346	76.2 (50.6, 115)

^*a*^ CI, confidence interval; GLSM, geometric least square mean.

^*b*^ "—", Not Appropriate.

**Fig 2:**
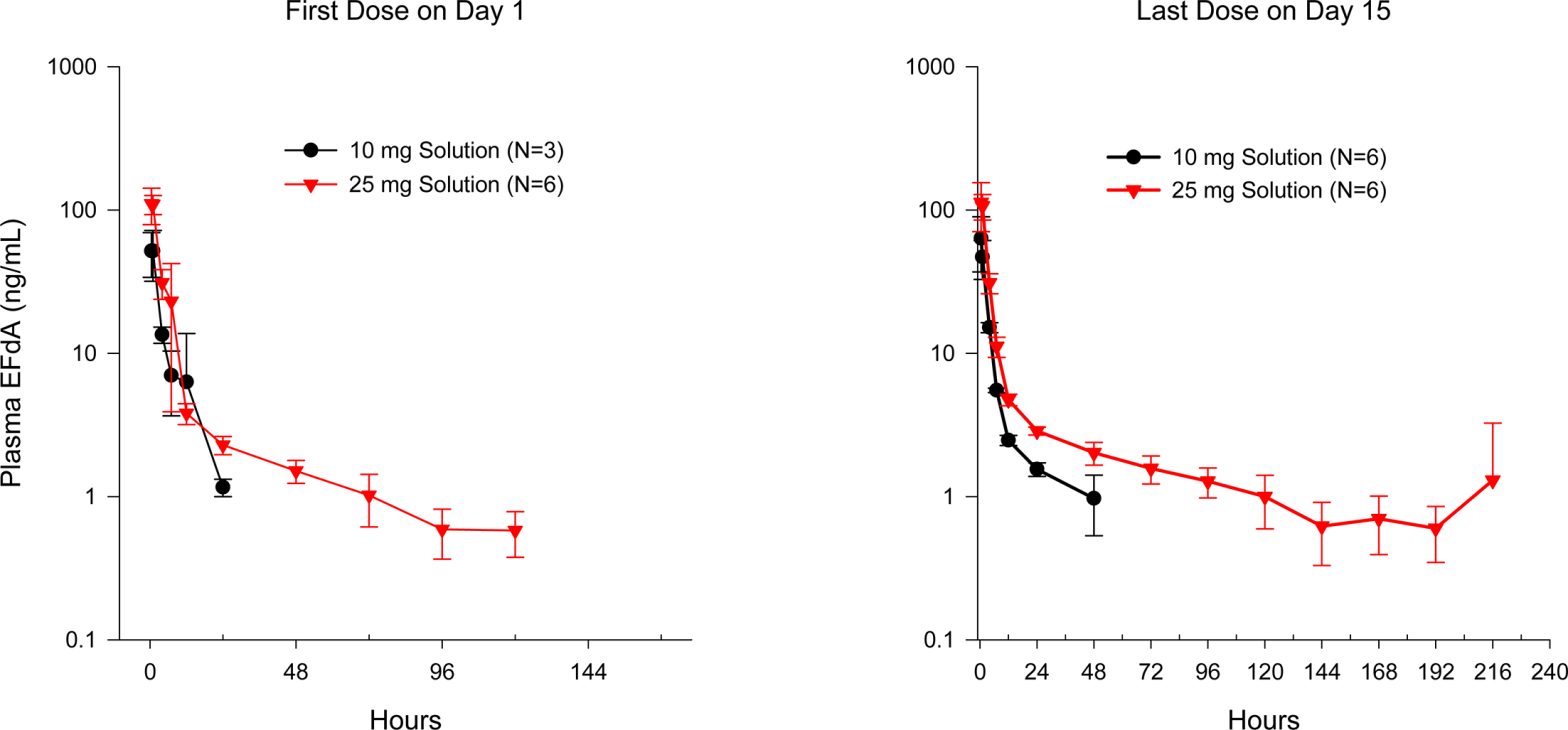
Mean (±SD) plasma EFdA concentrations following multiple ascending doses of BRII-732.

Following multiple-dose administration of BRII-732 at 10 mg or 25 mg, maximum EFdA plasma concentrations were achieved mostly around median *T*_max_ of 0.5 hours. PK profiles after three weekly doses of BRII-732 (Day 15) were comparable to those after the first dose on Day 1 (Fig. 2). Mean *t*_1/2_, where reportable, was approximately 31–75 hours. As measured by *C*_max_ and AUC_0-168h_, there was minimal accumulation of EFdA in plasma over three weekly doses of BRII-732 at 10 and 25 mg (Table 6).

**TABLE 6:** EFdA mean pharmacokinetic parameters in plasma following multiple ascending doses of BRII-732 (Study 1)^*a*^

	Weekly 10 mg solution		Weekly 25 mg solution	
Parameter	Day 1 (*N* = 3)	Day 15 (*N* = 3)	Day 1 (*N* = 6)	Day 15 (*N* = 6)
*C*_max_ (ng/mL)	55.6 (31)	63.5 (42)	120 (19)	121 (26)
*T*_max_ (h)	0.50 (0.5, 1.0)	0.5 (0.5, 1.0)	0.53 (0.5, 1.0)	0.5 (0.5, 1.0)
AUC_0-168h_ (h*ng/mL)	NC	295 (NC)^*b*^	577 (21)^*e*^	614 (10)
*C*_168h_ (ng/mL)	NC	NC	NC	1.1 (13)^*c*^
*t*_1/2_ (h)	NC	31.1 (NC)^*b*^	45.7 (45)^*e*^	75.4 (26)^*d*^
AR_Cmax_	—^*f*^	1.1 (18)	—	1.0 (16)
AR_AUC0-168h_	—	NC	—	1.1 (23)

^*a*^ Data are presented as mean (CV%); for *T*_max_, median (min, max) is reported. AR, accumulation ratio; NC, not calculable; due to unquantifiable concentrations at later timepoints, terminal phase-dependent parameters could not be calculated.

^*b*^ n=1.

^*c*^ n=2.

^*d*^ n=4.

^*e*^ n=5.

^*f*^ "—", Not Appropriate.

Between-subject variability in *C*_max_ and AUCs was low to moderate.

#### EFdA-TP in PBMCs

In Study 1, EFdA-TP concentrations were potentially underestimated due to sample handling issues and, therefore, were deemed unsuitable for quantitative assessments. Consequently, EFdA-TP PK parameters from Study 1 are not included in this paper; instead, the concentration profiles from Study 1 are only used for qualitative assessment, such as evaluating accumulation after multiple doses. Accurate sample handling and the addition of the appropriate amount of stabilizing agent are critical to prevent the rapid degradation of EFdA-TP. Lessons learned from Study 1 informed updates to PBMC sample stabilization procedures in Study 2, leading to improved collection and analysis of EFdA-TP. Thus, EFdA-TP PK parameters are presented for Study 2 only.

**Fig 3:**
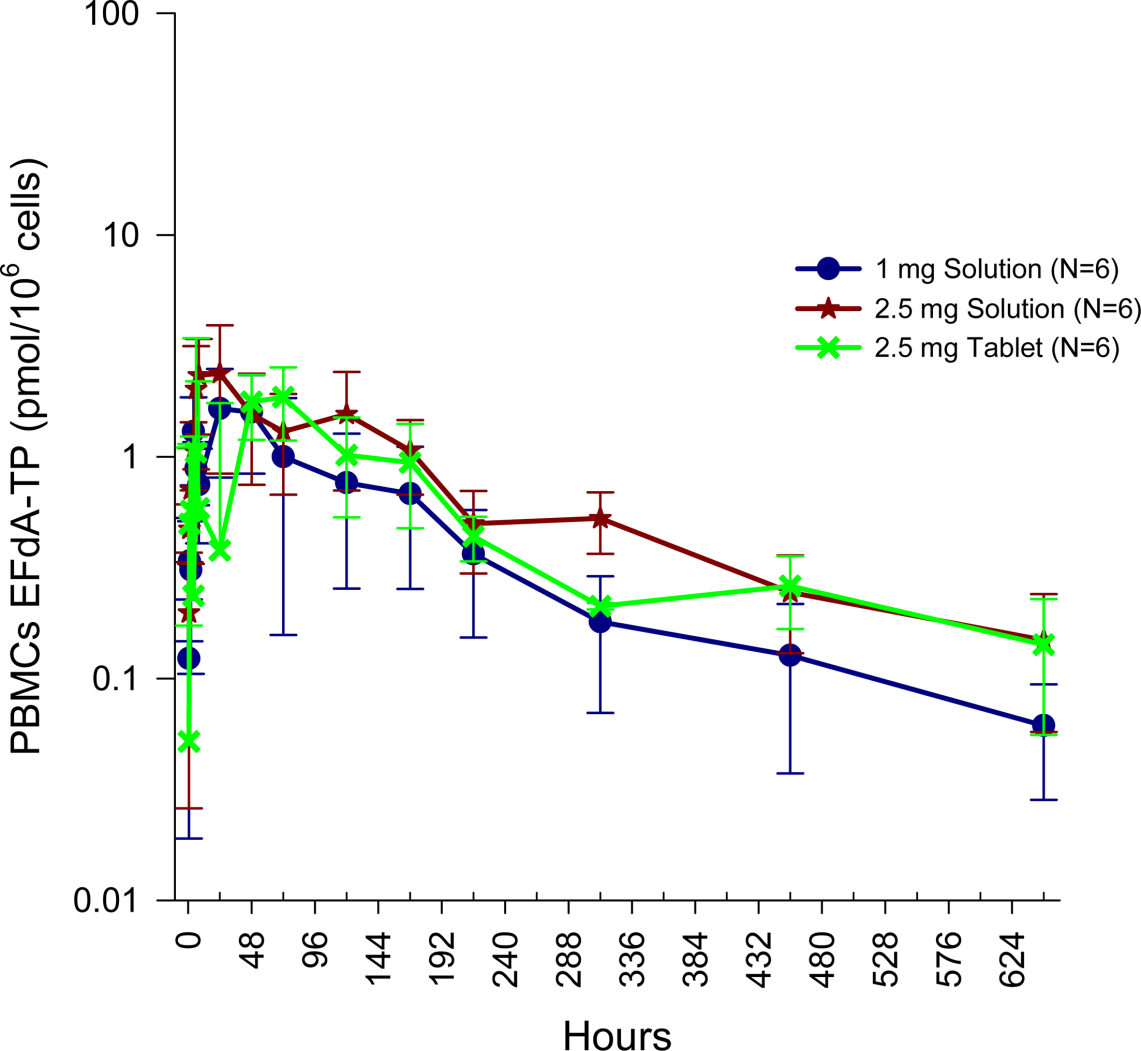
Mean (±SD) PBMCs EFdA-TP concentrations following single ascending doses of BRII-732.

In Study 2 after single oral administration of 1 or 2 mg BRII-732, EFdA-TP concentrations in PBMCs indicated rapid intracellular formation and subsequently slow cellular elimination (Fig. 3). Median *T*_max_ ranged from 6 to 14 hours. Following *C*_max_, EFdA-TP concentrations declined slowly. The median time of the last measurable concentration (*T*_last_) was 648 to 672 hours, and mean *t*_1/2_ was 194 to 227 hours (Table 7).

**TABLE 7:** EFdA-TP mean pharmacokinetic parameters in PBMCs following single ascending doses of BRII-732 (Study 2)^*a*^

Parameter	1 mg solution (*N* = 6)	2.5 mg solution (*N* = 6)	2.5 mg tablet (*N* = 6)
*C*_max_ (pmol/10^6^ cells)	1.89 (42)	2.94 (40)	2.65 (25)
*T*_max_ (h)	14.0 (4.0, 48.0)	6.0 (6.0, 24.0)	6.0 (6.0, 168.0)
AUC_0-last_ (h*pmol/10^6^ cells)	227 (52)	431 (37)	366 (19)
AUC_0-inf_ (h*pmol/10^6^ cells)	275 (43)^*b*^	479 (35)	348 (13)^*c*^
*T*_last_ (h)	648 (72.0, 648)	672 (432, 672)	648 (648, 648)
*t*_1/2_ (h)	201 (56)^*b*^	194 (38)	227 (39)^*c*^
*C*_168h_ (pmol/10^6^ cells)	0.683 (63)^*d*^	0.943 (49)	1.07 (37)

^*a*^ Data are presented as mean (CV%); for *T*_max_, median (min, max) is reported.

^*b*^ n=4.

^*c*^ n=3.

^*d*^ n=5.

Over the dose range of 1–2.5 mg BRII-732, PK exposure (*C*_max_ and AUCs) of EFdA-TP in PBMCs increased with dose; a 2.5-fold increase in dose resulted in an approximately 1.6-fold increase in *C*_max_ and 1.7- to 1.9-fold increase in AUCs. At 2.5 mg BRII-732 as tablet and/or solution, mean *C*_max_ was 2.65–2.94 pmol/10^6^ cells, and mean concentration at 7 days post-dose (*C*_168h_) was 0.68–1.07 pmol/10^6^ cells. Statistical analysis of PK exposure parameters showed that tablet and solution had comparable *C*_max_ and AUC_0-last_ values, with the ratio of GLSMs (90% CI) being 88.3% (65.3, 119) and 94.0% (65.4, 135), respectively. The AUC_0-inf_ for tablet appeared to be lower than for solution, with a ratio of GLSMs of 76.2% (Table 5). However, due to the limited sample size (*n* = 3) for AUC_0-inf_ from tablet formulation, the result should be interpreted cautiously.

Between-subject variability in *C*_max_ and AUCs was generally low to moderate.

## DISCUSSION

HIV patients have consistently stated a preference for approaches which could reduce the medication burden of life long once daily dosing, suggesting the importance of continuing to explore safe and effective options to achieve this goal for patients living with HIV. Once weekly BRII-732 could provide an important option for combination regimens which seek to reduce the daily pill burden for patients living with HIV.

Safety, tolerability, and pharmacokinetics have been evaluated at single and multiple doses of BRII-732 from two phase 1 studies. Results from these studies have demonstrated that BRII-732 was safe and well tolerated following single and multiple oral administrations of BRII-732 in healthy adult participants evaluating a wide range of doses.

Although not observed in the phase 1 studies with BRII-732 described here, a clear dose-response relationship has been established in chronic dosing studies with islatravir, which links increasing intracellular concentrations of EFdA-TP with lymphocyte apoptosis (12, 13). Specifically, doses or exposures that are equivalent to once daily islatravir 0.75 mg or above are likely to exhibit an impact on peripheral lymphocytes and associated lymphocyte sub-sets. This relationship has also established an exposure threshold, below which it is likely that islatravir can be safely dosed without a clinically meaningful impact on lymphocytes. Doses or exposures comparable to once daily islatravir 0.25 mg are not anticipated to cause lymphocyte damage at a mean population level (12). There is also sufficient clinical and modeling data supporting the predicted clinical efficacy of once daily islatravir 0.25 mg for the treatment of HIV, warranting evaluation in future clinical studies. These conclusions are supported by large clinical studies and data sets, enhancing confidence in the results (14). These Phase 1 studies explored the evaluation of a once weekly dose regimen of BRII-732 which would remain below exposures expected to impact peripheral lymphocytes, while remaining highly efficacious against HIV-1.

BRII-732, a pro-drug of EFdA, was rapidly and efficiently converted to EFdA by various hydrolytic enzymes, as evidenced by the rapid appearance of EFdA in plasma (median *T*_max_ of 0.5–1 hour) and the absence of measurable systemic exposure of BRII-732 throughout the sample collection period (Table 3). The PK exposure parameters of EFdA increased nearly proportionally with BRII-732 dose levels from both studies, indicating dose-linear PK characteristics (Table 4). Based on the observed *t*_1/2_ values of EFdA and the weekly dosing regimen, significant accumulation of EFdA in plasma was not anticipated. The observed results after three weekly doses of BRII-732 at 10 and 25 mg are consistent with the prediction (Table 6).

After the administration of BRII-732, efficient intracellular formation of EFdA-TP was observed, along with the anticipated slow cellular elimination. The median *T*_max_ ranged from 6 to 14 hours and mean *t*_1/2_ was between 194 and 227 hours (Table 7). Based on the observed mean *t*_1/2_ values of EFdA-TP and the weekly dosing regimen of BRII-732, an approximately 2.2- to 2.5-fold accumulation is anticipated.

The EFdA-TP PK profile after oral administration of BRII-732 was similar to profiles reported in literature for islatravir (15–17), which allows leveraging the target coverage established for islatravir. Published results from islatravir clinical studies and related PK/PD assessments suggest that a steady-state intracellular EFdA-TP trough concentration of ≥0.05 pmol/10^6^ cells is required for efficacy, and once daily islatravir at doses of 0.25 and 0.75 mg achieved average EFdA-TP trough concentration of approximately 1.6 pmol/10^6^ cells and 6.3 pmol/10^6^ cells, respectively, which were associated with effective clinical antiviral activity (14, 17). Additionally, preclinical studies in rhesus macaques demonstrated that once weekly dosing of islatravir achieving PBMC islatravir-triphosphate concentrations of ≥0.53 pmol/10^6^ cells was sufficient to maintain HIV-1 viral suppression (18).

In this study, single doses of 1–2.5 mg BRII-732 administered as oral solution or tablet resulted in mean intracellular EFdA-TP concentrations at 7 days post-dose (trough concentration) of 0.68–1.07 pmol/10^6^ cells. Considering the anticipated two- to threefold accumulation factor of intracellular EFdA-TP, the steady-state trough concentration of EFdA-TP after once weekly oral administration of BRII-732 would be approximately 1.4–3.2 pmol/10^6^ cells. These concentrations fall within the established therapeutic window for islatravir, warranting the continued clinical investigation of BRII-732 as a once weekly dosing option in HIV-infected patients.

## SUPPLEMENTAL MATERIAL

Fig. S1 aac.00200-25-s0001.docx Chemical structures of EFdA and BRII-732.

ASM does not own the copyrights to Supplemental Material that may be linked to, or accessed through, an article. The authors have granted ASM a non-exclusive, world-wide license to publish the Supplemental Material files. Please contact the corresponding author directly for reuse.
