# Supplementary material for: Two phase 1 studies of safety, tolerability, and pharmacokinetics of an EFdA prodrug (BRII-732) in healthy adult participants
Source: Antimicrob Agents Chemother. 2025 Apr 22;69(6):e00200-25. doi: 10.1128/aac.00200-25 (PMC12135525; doi:10.1128/aac.00200-25)
Supplement: Fig. S1 — Chemical structures of EFdA and BRII-732. [file aac.00200-25-s0001.docx]

**SUPPLEMENTAL MATERIALS**

Two Phase 1 Studies of Safety, Tolerability, and Pharmacokinetics of an EFdA prodrug (BRII-732) in Healthy Adult Participants

David Margolis^1#^, Michael Watkins^1^, Yali Zhu^1^

1 Brii Biosciences, Inc., Durham, NC, United States

**Figure S1: Chemical structures of EFdA and BRII-732**
